# Emeritus Professor Samuel Ofosu-Amaah (1931-2023)

**DOI:** 10.4314/gmj.v57i1.1

**Published:** 2023-01

**Authors:** David Ofori-Adjei

**Figure:**
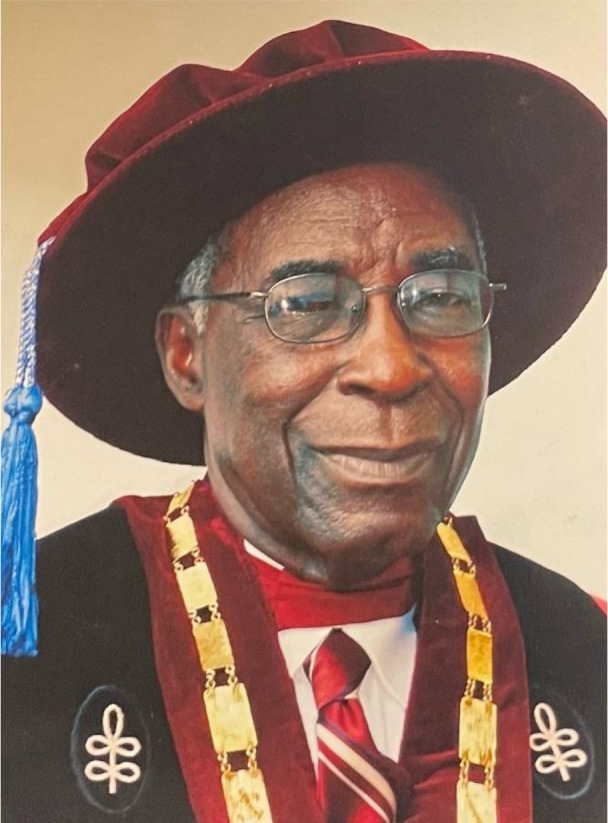


Emeritus Professor Samuel Ofosu-Amaah, who died on 22 January 2023, was highly regarded in academia, medical education, and public health. Trained as a paediatrician, he built a significant niche in Community Health early in his career at the University of Ghana Medical. A review of his career in the last half-century reveals a man best described as a visionary leader.^1^

The words of Ralph Waldo Emerson, *“Do not follow where the path may lead. Go instead where there is no path and leave a trail”,* are well suited to the life of Emeritus Professor Samuel Ofosu-Amaah.

Dr Anarfi Asamoah-Baah (Former Deputy Director-General of the World Health Organisation and alumnus of the University of Ghana Medical School) said this about him: “*Prof [Ofosu-Amaah] was a rare cocktail of brilliance, intellectual dexterity, softness, dogged determination and foresight, stammering eloquence, compassion and respect, tolerance*”.

Similar expressions have resonated in tributes since the announcement of his passing. His gentle approach to dealing with everyone and every challenge is outstanding among the tributes. Many of his students and colleagues saw him as the epitome of a mentor.

Professor Ofosu-Amaah loved to share knowledge. He organised lectures on the history of medicine for medical students.

One of his closest colleagues in the establishment of the School of Public Health, Dr Nana Amuasi Enyimayew, said this of him: *“He knows everything from archaeology to zoology. Having a conversation with him was a great delight”.*

At a time when computers were not common gadgets in medical research and practice, Professor Ofosu-Amaah demonstrated a keen interest and knowledge of their use more than his younger colleagues and faculty.

One of Professor Ofosu-Amaah's public health passions was sanitation. In the 2005 J.B Danquah Memorial Lectures at the Ghana Academy of Arts and Sciences, he laments the ignorance of the Ghanaian of the “germ theory” of disease causation and the filthy state of our environment. He was appointed a member of the Accra Metropolitan Assembly and chaired the Health Committee of the Assembly. Sanitation was one of his major concerns. His contributions were directed at making “Accra clean again”. Many of the suggestions made during his time are now being implemented to improve sanitation in Accra.

His major international contributions to health include working with the World Health Organisation, serving as a Senior Adviser in Health at UNICEF, and being a member of the Panel on International Health of the United States Pharmacopoeia, Division of Drug Information and the World Bank. For the decade he spent at UNICEF, he significantly contributed to implementing the Bamako Initiative, a project to improve access to medicines in resource-poor countries.

At home in Ghana, his footprints are indelible in many landmark schemes. These include co-director of the Danfa Comprehensive Rural Health and Family Planning Project (Danfa Project), Foundation Director of the University of Ghana School of Public Health, the First President and Chairman of the Council of the Ghana College of Physicians and Surgeons, the University for Development Studies and the Board of Korle Bu Teaching Hospital,

Those who worked with him recall how he used “soft power” to drive his agenda. Dr Nana Amuesi Enyimayew surmises his attitude to work as portraying that “*..humility, gentility and an acceptance of everybody actually enhance but not diminish a great person*”.

The three health training institutions he was involved in have all grown to attain full stature and recognition and will continue to respond to national needs for generations to come. He demonstrated visionary leadership and “*clear patriotism in all he did*”, according to Dr Delanyo Dovlo, Director of Human Resources at the Ministry of Health, during the establishment of the School of Public Health.

The Danfa Project and his study on lameness in school children are recognised as significant driving factors in the global “Health for All” and Universal Health Coverage movement and the drive towards mass immunisation campaigns for poliomyelitis control and elimination.

Emeritus Professor Samuel Ofosu-Amaah received several citations and awards for his contribution to humanity. The Republic of Ghana awarded him the state honour of Member of the Order of the Volta in 2006.

On 24 August 2022, the Ghana College of Physicians and Surgeons, in collaboration with the Ministry of Health and the Ghana Health Service, organised a symposium in his honour and awarded him the “Outstanding National and Global Leadership in Health Award 2022”.

Emeritus Professor Ofosu-Amaah will be remembered as a pillar in the medical profession, a trailblazer, an example of dedication to duty, and a Christian gentleman.

He is survived by his wife, Virginia, three children and grandchildren.

## References

[1] Documentary-Professor Samuel Ofosu-Amaah Ghana College of Physicians and Surgeons 2022.

